# Further assessment of *Salvia haenkei* as an innovative strategy to counteract skin photo-aging and restore the barrier integrity

**DOI:** 10.18632/aging.202464

**Published:** 2021-01-08

**Authors:** Veronica Cocetta, Jessica Cadau, Miriam Saponaro, Isabella Giacomini, Stefano Dall’Acqua, Stefania Sut, Daniela Catanzaro, Genny Orso, Giorgia Miolo, Luca Menilli, Andrea Pagetta, Eugenio Ragazzi, Monica Montopoli

**Affiliations:** 1 Department of Pharmaceutical and Pharmacological Sciences, University of Padova, Padova 35121, Italy; 2 Veneto Institute of Molecular Medicine (VIMM), Padova 35121, Italy; 3 Department of Medicine, University of Padova, Padova 35128, Italy

**Keywords:** skin aging, skin photoaging, *Salvia haenkei*, skin barrier integrity, wound healing

## Abstract

Skin is the essential barrier of the human body which performs multiple functions. Endogenous factors, in concert with external assaults, continuously affect skin integrity, leading to distinct structural changes that influence not only the skin appearance but also its various physiological functions. Alterations of the barrier functions lead to an increased risk of developing disease and side reactions, thus the importance of maintaining the integrity of the epidermal barrier and slowing down the skin aging process is evident.

*Salvia haenkei* (SH) has been recently identified as a potential anti-senescence agent; its extract is able to decrease the level of senescent cells by affecting the IL1α release and reducing reactive oxygen species (ROS) generation.

In this study, SH extract was tested on human keratinocyte cell line (HaCaT) exposed to stress factors related to premature aging of cells such as free radicals and ultraviolet B radiation. We confirmed that SH acts as scavenger of ROS and found its ability to restore the skin barrier integrity by reinforcing the cytoskeleton structure, sealing the tight junctions and increasing the migration rate of cells.

Given these results, this work becomes relevant, identifying *Salvia haenkei* as a compound useful for anti-aging skin treatment in clinical performance.

## INTRODUCTION

Healthy and functioning skin is essential to protect against dehydration and penetration of various microorganisms, allergens, irritants, and radiation. The daily exposure to extrinsic agents combined with some intrinsic factors, such as genetics, cellular metabolism, hormone and metabolic processes, leads to the natural aging of the skin [1]. In particular, skin’s aging phenomenon is hugely related to ultraviolet radiation, pollution, repetitive muscle use, and gravity [2, 3]. Skin aging therefore results from cumulative structural and physiological alterations and progressive modulation of skin layers [2, 4, 5]. Even if aging is not considered a pathological condition, but rather a biological and inevitable process, its correlation with a variety of skin diseases is now well established. Considering that the most relevant age-related skin disease are degenerative disorders, benign and malignant neoplasms, etc., the importance of skin caring to treat or prevent certain cutaneous disorders results to be evident. [6–8].

Nowadays, skin anti-aging strategies act reversing dermal and epidermal signs of photo/chronological-aging. Based on their approaches, they can be classified as follows: cosmetological care, topical agents, invasive procedures, systemic agents, avoiding of aging exogenous factors, correction of lifestyle and habits, preventive medicine [9]. There are two main groups of agents that can be used as components of anti-aging formulations: 1. Cell regulators, such as retinols, peptides and growth factors, directly acting on collagen metabolism and production; 2. Antioxidants, like vitamins, polyphenols, flavonoids, able to reduce collagen degradation [9].

Substantial evidence supports the idea that skin aging is mainly driven by ROS, generated during endogenous oxidative cell metabolism [10, 11]. It is known that ROS accumulation is responsible for cell damage including lipid peroxidation, membrane protein damage and DNA mutation that lead to structural and functional skin alterations. In the course of time, human cells underpin a natural increase of ROS levels, associated with partial loss of the antioxidant defenses. This inevitable condition is further fostered by daily ultraviolet irradiation (UVR) that induces matrix metalloproteinases (MMPs) synthesis and, consequently, MMP-mediated collagen destruction [12, 13]. *In vivo* studies demonstrated the causal relationship between mitochondrial oxidative damage, cellular senescence, and aging phenotypes [10, 14].

Cellular senescence is defined as a stable arrest of cell growth that occurs in all human cells during aging [15–17]. As a result of stressful events, such as oncogene over-expression, ROS generation and DNA damage (for example induced by UVR, in particular by UVB light), cells may prematurely become senescent [18]. In this scenario, it appears evident the importance of developing innovative strategies able to prevent the accumulation of senescent cells or to selectively kill them, thus counteracting aging and aging-associated disorders. Recently, we identified *Salvia haenkei* (SH), a Bolivian plant rich in vitamin B and antioxidant properties, as a potential anti-senescence agent [18].

The accumulation of senescent cells over time is well known to be one of the most important mechanisms through which tissues undergo the ageing process, which appears to be principally triggered by the persistence of a typical inflammatory status. In fact, despite senescent cells are unable to proliferate, they possess high metabolic activity and they efficiently produce several pro-inflammatory molecules [19]. By using a model of skin human epidermis (EpiSkin), it was indeed demonstrated that SH extract decreases the levels of senescent cells by affecting the IL1ɑ release and reducing ROS generation [18].

Under homeostatic conditions, the skin is an efficient barrier that protects from external assaults and endogenous factors, and regulates the loss of fluid, electrolytes, and proteins. This important task is mainly ascribed to the epidermis, a dynamic, highly stratified epithelium. The two major epidermis constituents are stratum corneum, the external layer, and tight junctions (TJs), intercellular junctions that seal adjacent keratinocytes in the stratum granulosum [20]. Cell–cell adhesions are necessary to maintain the epidermal-barrier integrity. This important function is fulfilled by specialized cell–cell junctions: adherens junctions (AJs), desmosomes, and tight junctions. These structures not only permit physical connections between cells, but overall organize cytoskeletal elements, modulate signaling pathways involved in tissue development, structure, and physiology. Recent genetic studies unexpectedly revealed a non-canonical role of junctional proteins, bringing out the importance of junctional crosstalk, interdependencies, and compensation to allow tissue robustness [21].

Here we studied the activity of a *Salvia haenkei* extract to understand the molecular mechanism responsible for its protective effects on photo/chronological skin aging. With this perspective, human spontaneously immortalized keratinocytes (HaCaT) were used as *in vitro* model, and SH mechanism of action was analyzed under basal regimen as well under stressful condition induced by H_2_0_2_ and/or UVB irradiation.

## RESULTS

### Safe profile of SH extract and antiaging properties

Oxidative stress in skin plays a major role in the aging process and in the pathogenesis of several skin disorders [22]. Matic et al. already demonstrated the anti-senescence activity of *Salvia haenkei* also correlated to its scavenging properties [18]. These protective effects might be exploited to develop a novel skin-repairing treatment able to preserve the integrity of the barrier. At the aim of deeply investigate SH properties, the extract activity on cell viability and ROS generation in HaCaT cell model was firstly verified.

**Figure 1:**
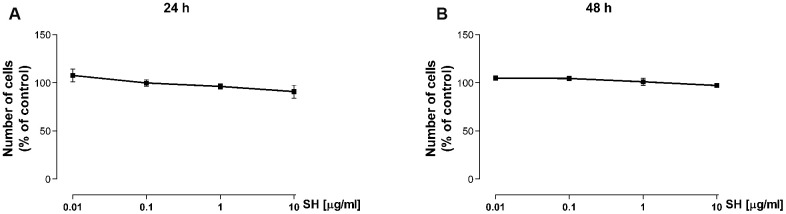
Effect of *Salvia haenkei* extract on HaCaT cell viability after 24 (**A**) and 48 (**B**) hours of treatment. Results are expressed as percentage of cell number compared to control (mean ± SEM of 3 independent experiments).

The cell viability assay was performed to address possible cytotoxic effects of the *Salvia haenkei* extract on keratinocytes. HaCaT cells were treated with SH extract (0.01–10 μg/ml) for 24 and 48 hours. Cell viability was assessed by Trypan blue Exclusion Assay. Figure 1A, 1B shows that treatment with SH extract does not affect cell viability confirming the safe profile of the extract on HaCaT cells at the used experimental conditions.

**Figure 2:**
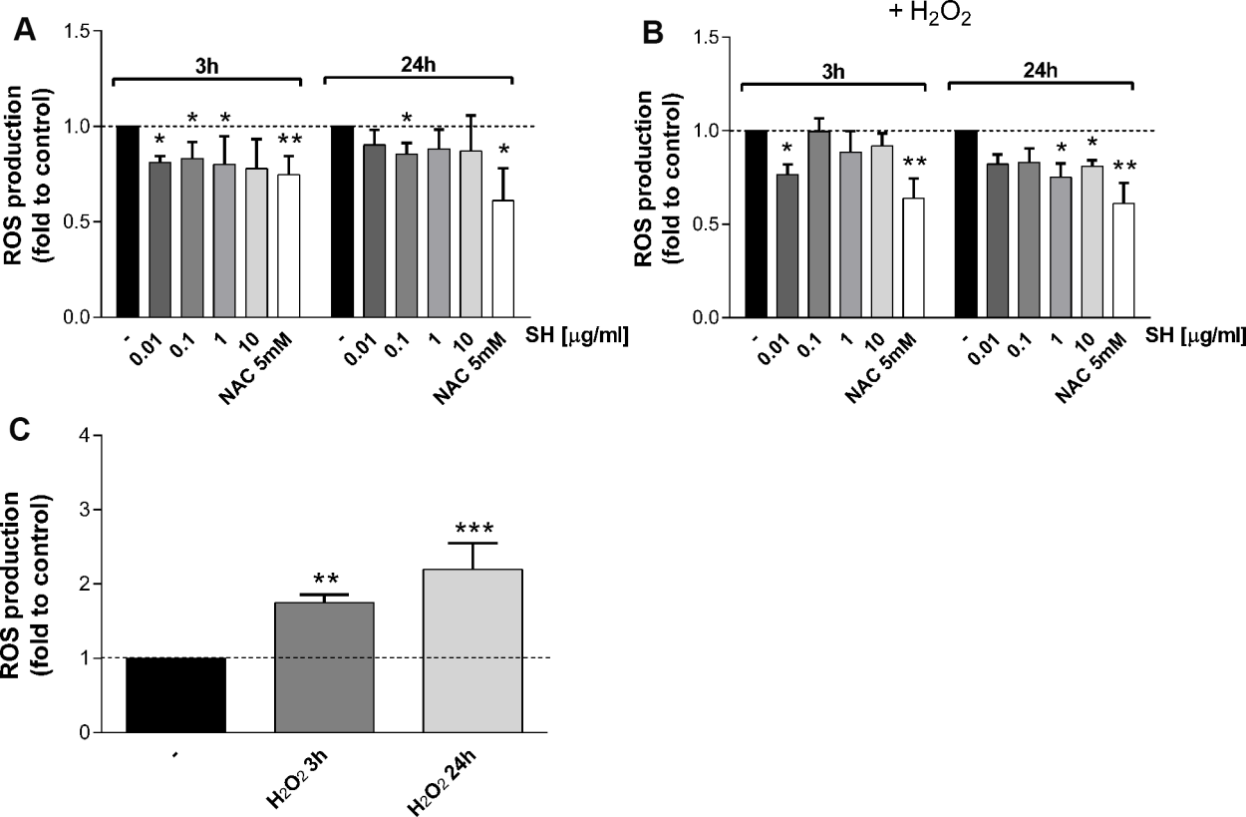
**Effect of *Salvia haenkei* extract 0.01-0.1-1-10 μg/ml on ROS generation in HaCaT cells in basal and oxidative stress conditions.** ROS were measured after 3-24 hours of incubation with SH under basal condition (**A**) or after H_2_O_2_ exposure (**B**, **C**). Figure (**C**) represents the effect of H_2_O_2_ stimulation of untreated cells, while figure (**B**) represents the effect of H_2_O_2_ stimulation of treated cells. NAC 5 mM is used as positive control. Data are expressed as mean ± SEM of fluorescence intensity (FI) of treated cells related to control. n = 3-4 experiments. *p<0.05, **p<0.01, ***p<0.001 treatment *vs* control.

The activity of *Salvia haenkei* extract on ROS generation was assayed in HaCaT cells after 3-24 hours of treatment and before and after H_2_O_2_ exposure. Results in Figure 2A show that SH is able to reduce basal ROS levels within the cells, particularly for the 3 hour-treatment time. Besides a significant increase in ROS levels after H_2_O_2_ stimulation (Figure 2C), SH is able to prevent the production of ROS. SH extract (0.01 μg/ml) significantly reduced (-25%) ROS generation induced by the oxidative stimulus (Figure 2B), showing an effect comparable with NAC after 3 hours of treatment. 24 hours of SH treatment appear to be more effective in counteracting the ROS increment, significantly reducing ROS levels at 1 and 10 μg/ml (Figure 2B). These results confirm *Salvia haenkei* antioxidant effects, as previously observed [18], showing an antioxidant basal effect already starting from short treatments; the effect in preventing oxidative stimulus-induced increase is most relevant for longer treatment times.

**Figure 3:**
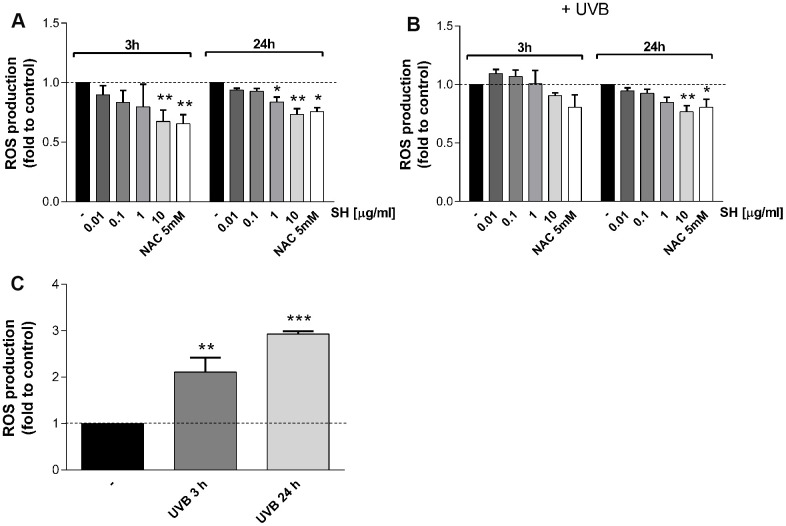
**Effect of *Salvia haenkei* extract 0.01-0.1-1-10 μg/ml on ROS generation in HaCaT cells in basal and UVB-stimulated conditions (30 KJ/m^2^).** ROS were measured after 3-24 hours of incubation with SH under basal condition (**A**) and after UVB exposure (**B**, **C**). Figure (**C**) represents the effect of UVB stimulation of untreated cells, while figure (**B**) represents the effect of UVB stimulation of treated cells. NAC 5 mM is used as positive control. Data are expressed as mean ± SEM of fluorescence intensity (FI) of treated cells related to control. n = 3-4 experiments. *p<0.05, **p<0.01, ***p<0.001treatment *vs* control.

It is reported that ultraviolet radiations induce increase in intracellular level of ROS in human skin causing damages and premature aging and other pathologies [23–25]. Therefore, the effect of SH extract on UVB-mediated ROS production has been evaluated. As expected, results in Figure 3C show a significant increase in intracellular ROS level following UVB stimulation. Treatment with SH, which induces a tendency to reduce basal ROS levels within the cells (Figure 3A), is able to prevent the increase induced by UVB stimulation (Figure 3B). 3 hours of SH treatment induce a tendency to reduce ROS levels; a longer SH treatment is more effective in counteracting the oxidative stress, showing significant decrease as reported in Figure 3A. These data underline the antioxidant activity of *Salvia haenkei* also in UVB mediated oxidative stress.

**Figure 4:**
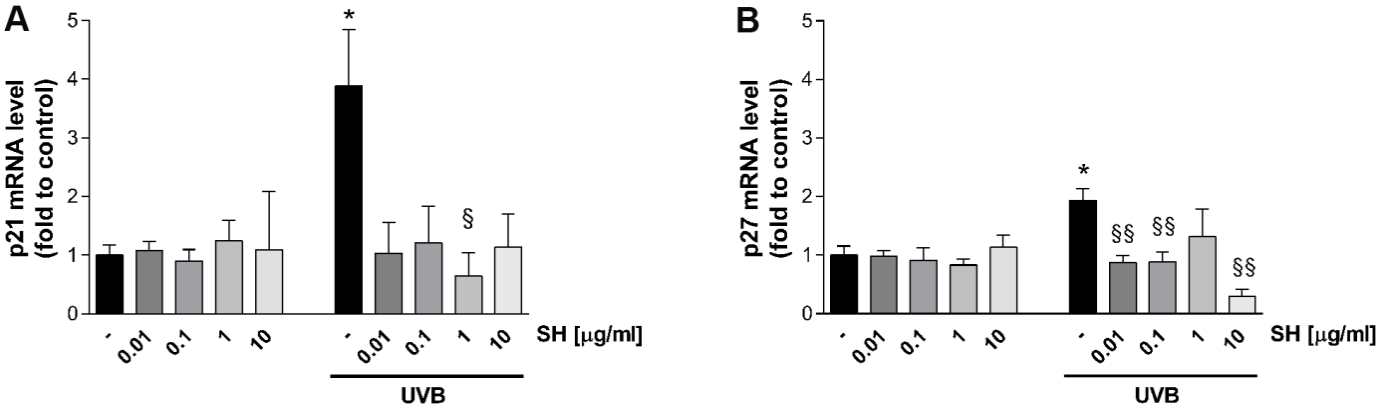
Effect of *Salvia haenkei* extract 0.01-0.1-1-10 μg/ml on p21 (**A**) and p27 (**B**) mRNA levels in HaCaT cells exposed and not exposed to UVB radiation (30 KJ/m^2^). Results are normalized to GAPDH, expressed as ratio of treatment *vs* control not exposed to UVB, and are the mean ± SEM of 3 independent experiments. *p < 0.05, treatment *vs* control-UVB; §*p* < 0.05, §§*p* < 0.01treatment *vs* control+UVB.

As mentioned, increasing evidence suggests that senescent cells accumulate in chronologically and photo-aged skin, contributing to age related-skin alterations and pathologies [26]. The effect of *Salvia haenkei* was therefore evaluated on p21 and p27 senescence markers (which prevent phosphorylation of the retinoblastoma protein (Rb) and with inhibitory activity on CDK2-cyclin E, respectively [27]) after photo-stress induced by ultraviolet radiation. Results in Figure 4 show that UVB irradiation induces a significant increase in p21 and p27 mRNA levels as compared to the unstimulated control. A similar trend in prevention of increase is observed in cells treated with SH. SH 1 μg/ml induces a significant reduction of p21 levels compared to UVB stimulated control, while the other concentrations induce a tendency to reduce (Figure 4A). P27 levels are significantly reduced by pretreatment with SH 0.01, 0.1 and 10 μg/ml (Figure 4B).

**Figure 5:**
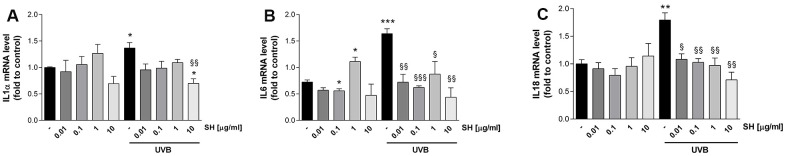
Effect of *Salvia haenkei* extract 0.01-0.1-1-10 μg/ml on IL1α (A), IL6 (B) and IL18 (B) mRNA levels in HaCaT cells exposed and not exposed to UVB radiation (30 KJ/m^2^). Results are normalized to GAPDH, expressed as ratio of treatment *vs* control not exposed to UVB, and are the mean ± SEM of 3 independent experiments. *p < 0.05, **p < 0.01, ***p < 0.001 treatment *vs* control-UVB; §*p* < 0.05, §§*p* < 0.01, §§§*p* < 0.001treatment *vs* control+UVB.

It is also established that senescent cells negatively affect the microenvironment by secreting a pro-inflammatory mixture of chemokines, cytokines, growth hormones and proteases, known as senescence-associated secretory phenotype (SASP) [28]. Results in Figure 5 show that UVB stimulation induces a significant increase in IL1α (A), IL6 (B), and IL18 (C) mRNA levels with respect to the unstimulated control. Pretreatment with SH presents a strong activity in counteracting the increase in cytokine levels. As shown in Figure 5A, SH 10 μg/ml significantly decreased IL1α levels; the increase in IL6 is significantly counteracted by SH 0.01,0.1 and 10 μg/ml (Figure 5B) while all the tested concentrations are able to significantly reduce IL18 levels (Figure 5C).

### Skin barrier maintenance

Given the above reported results on an epithelial cell line, the effect of SH extract on several proteins and processes involved in skin barrier maintenance and tissue damage repair was evaluated.

### Sirtuin-1 expression

**Figure 6:**
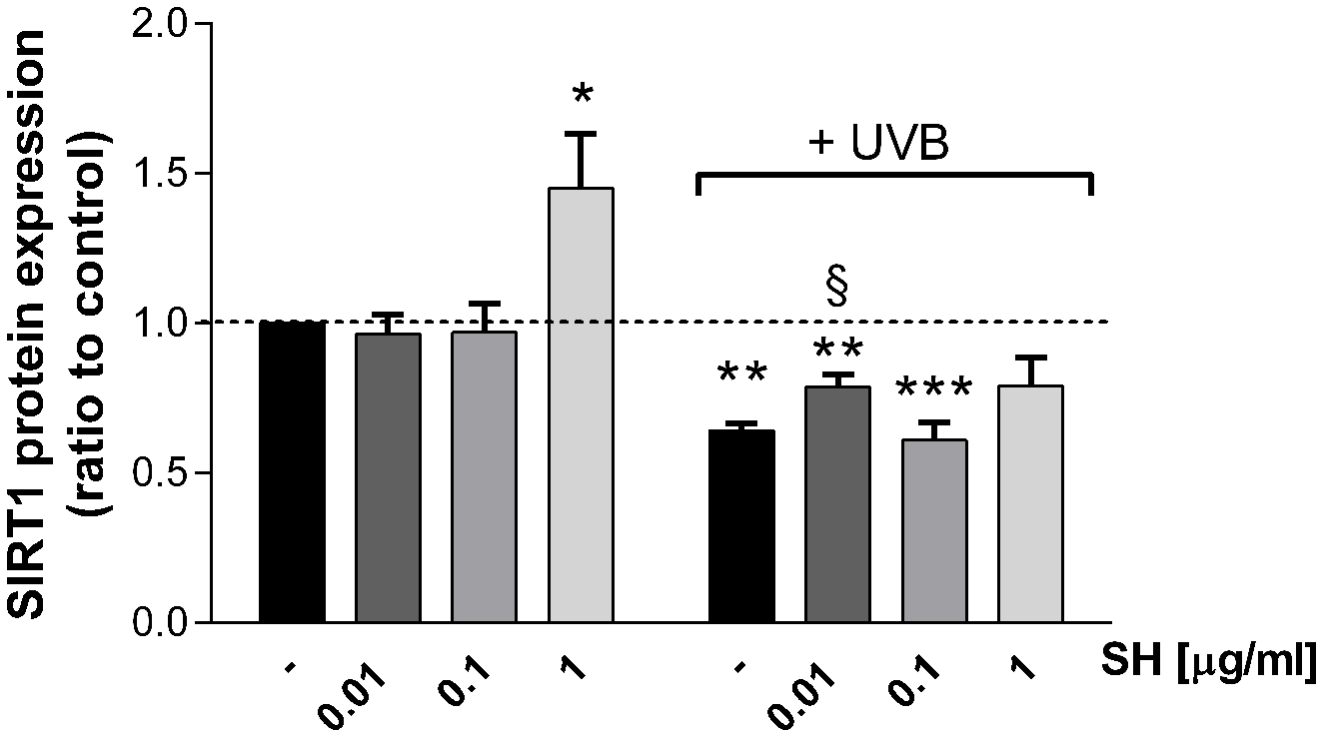
**Effect of *Salvia haenkei* extract 0.01-0.1 and 1 μg/ml on Sirtuin-1 protein expression in HaCaT cells exposed and not exposed to UVB radiation (30 KJ/m^2^).** Results are normalized to calnexin, expressed as a ratio of treatment *vs* control not exposed to UVB [+ UVB/ (ctr - UVB)], and are the mean ± SEM of 4 independent experiments. *p < 0.05, **p < 0.01, ***p < 0.001 treatment *vs* control-UVB; §*p* < 0.05, treatment *vs* control+UVB.

Several *in vitro* studies reported that UVB radiations induce a decrease in Sirtuin1 (SIRT1) protein levels of human fibroblasts, indicating an involvement of SIRT1 in the UVB-mediated damage [29]. So, the effect of *Salvia haenkei* extract on Sirtuin1 protein expression was evaluated by western blot assay (Figure 6). As expected, UVB stimulation induced a significant decrease in SIRT1 protein expression in HaCaT cells. The activity of SH extract on SIRT1 levels was therefore investigated. Interestingly, results show that 1 μg/ml SH extract, as compared to control, induced an increase of SIRT1 expression (+40%) under basal conditions, indicating a potential stimulating-effect on SIRT1 levels. As expected, UVB stimulation induces a significant reduction of SIRT1 levels. Results show that the pretreatment with 0.01 μg/ml SH extract exhibits ameliorative effect in Sirtuin 1 levels, as compared to the UVB-treated control.

### Wound healing assay

**Figure 7:**
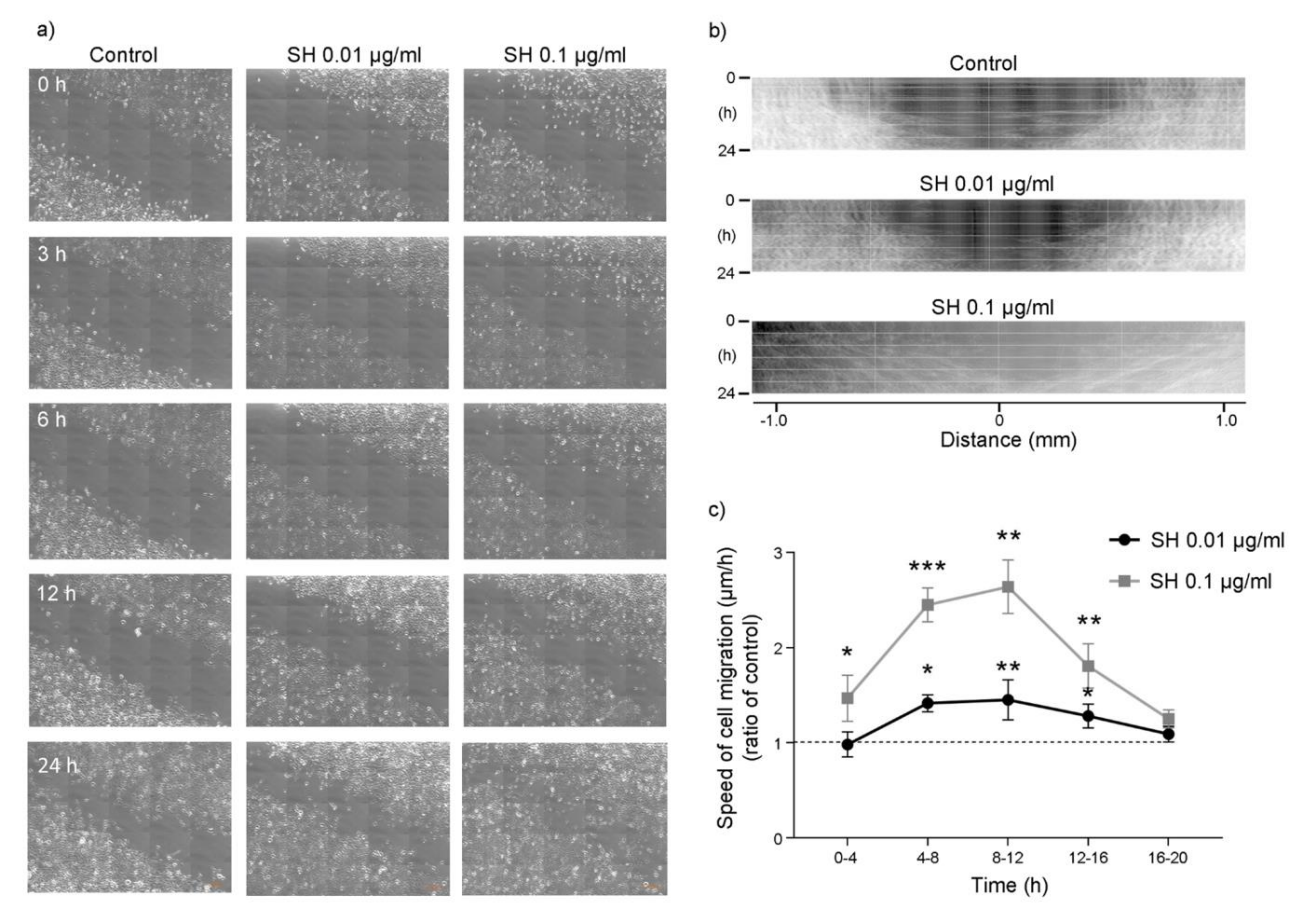
**Effect of *Salvia haenkei* extract on HaCaT cell migration - wound healing assay.** (**A**) Representative images showing the scratch (wound) at t = 0-3-6-12-24 h after treatment with 0.01-0.1 μg/ml SH. Images were acquired by confocal microscopy LSM 800 Software ZEN 2.1 Blue edition, 20X magnification and the scratch rate during the time was analyzed with the dedicated software. Figure (**B**) shows kymograms of HaCaT cells migration; (**C**) quantification of cells migration speed expressed as ratio of SH treatment to control (μm/h). Data are representative of 3 different experiments. *p<0.05, **p<0.01, ***p<0.001 treatment *vs* control.

Keratinocytes are the main constituents of epidermis, providing a barrier between the organism and the environment. Therefore, their proliferation and migration play a key role in the maintenance of an effective barrier and in wound healing following injuries [30, 31]. Thus, the effect of SH on HaCaT cell migration was evaluated. Images in Figure 7A show that treatment with SH leads to a reduction in the thickness of the scratch indicating an effect on HaCaT cell migration. Figure 7B reports kymograms construction of the migration velocity of HaCaT cells; images show a concentration-dependent increase in cells migration. Graph in Figure 7C indicates a significant increase in cells migration speed after 4-8 hours of treatment with 0.01 μg/ml SH extract. The maximum increase is reached in the 8-12 hours interval, where the rate in cell migration speed of cells is about 165% with respect to the control. These data, as the results obtained on cell proliferation, indicate that the lowest concentration of SH extract is able to induce an increase in cell migration speed not related to a hyperproliferative phenotype.

### Tight junctions’ maintenance

As well as stratum corneum, tight junctions (TJs) play an important role in skin integrity, acting as a barrier for water and solutes, and being involved in differentiation, proliferation, cell polarity, and signal transduction processes of cells. Recent studies demonstrated that UV-light alters expression and localization of single proteins of TJ structure, which in turn disrupts the barrier function of skin [32].

**Figure 8:**
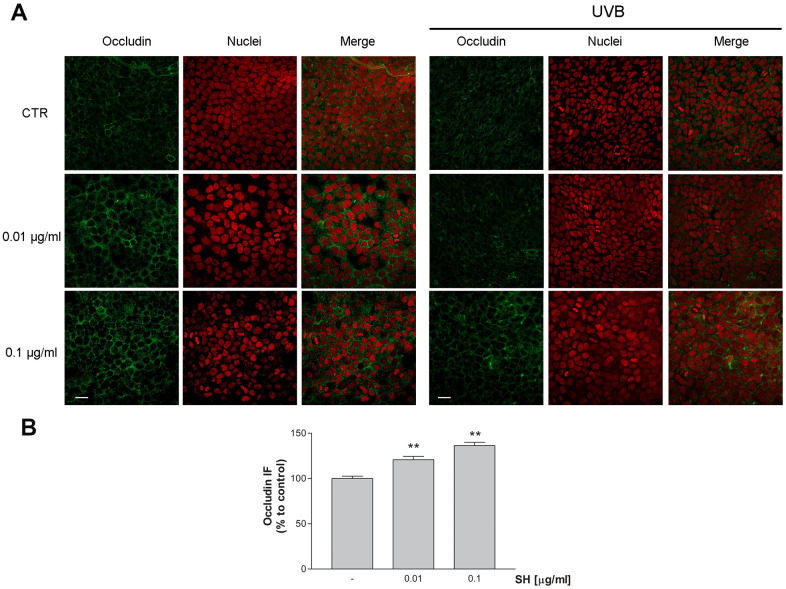
**Effect of *Salvia haenkei* extract 0.01 and 0.1 μg/ml on occludin in HaCaT cells before and after exposure to UVB radiation (30KJ/m^2^).** (**A**) Images were collected by confocal laser-scanning microscope LSM800 and software ZEN 2.1, magnification 60X and are representative of at least 3 experiments. Scale bar = 20μm. (**B**) Quantitative analysis of occludin fluorescence intensity (related to area) in cells treated with SH. Results are the mean ± SEM of 3-5 experiments. **p<0.01, treatment *vs* control.

In order to evaluate *Salvia haenkei* extract’s effect as possible protective agent against TJs disruption, expression of occludin was measured by immunocytochemistry following exposure to UVB radiation. Images in Figure 8A, 8B show that treatment with both concentrations of SH induces an increase in occludin protein expression, promoting junction’s formation useful to the maintenance of the epidermal barrier integrity. As expected, following exposure to UVB radiation, cells show an irregular and not defined membrane ring (Figure 8A). SH treatment, especially at the higher concentration, preserves the morphology of the membrane maintaining the structure comparable to the unstimulated control. SH extract promotes junction’s reinforcement and prevents the damage induced by UVB exposure, maintaining the functionality of the skin barrier.

### Filaggrin expression

**Figure 9:**
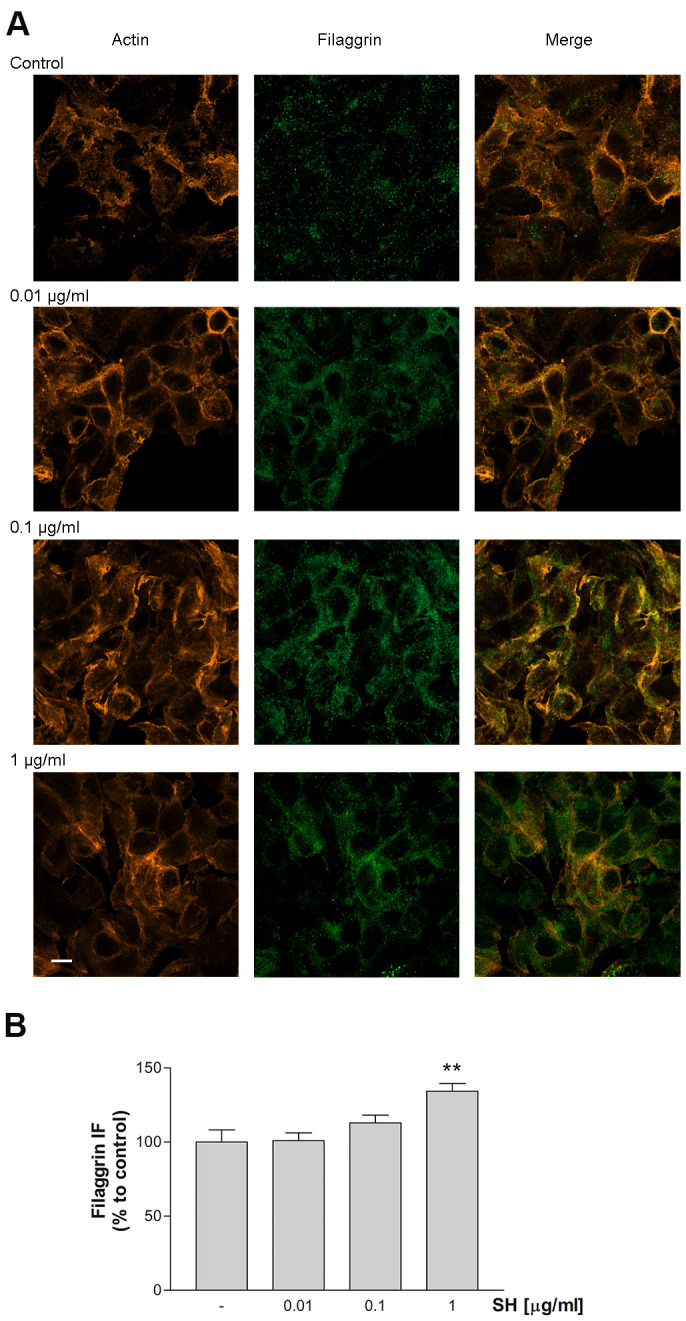
**Effect of *Salvia haenkei* extract 0.01- 0.1 and 1 μg/ml on filaggrin and actin proteins expression in HaCaT cells.** (**A**) Images were collected by confocal laser-scanning microscope LSM800 and software ZEN 2.1, magnification 60X, and are representative of at least 3 experiments. Scale bar = 10μm. (**B**) Quantitative analysis of filaggrin fluorescence intensity (related to area). Results are the mean ± SEM of 3-5 experiments. **p<0.01, treatment *vs* control.

The effect of *Salvia haenkei* extract on filaggrin (FLG) protein expression was evaluated by immunocytochemistry. Filaggrin is a structural protein fundamental in the development and maintenance of the skin barrier. Results in Figure 9A, 9B show a concentration-dependent increase in filaggrin protein expression following treatment for 24h with SH extract. Furthermore, the expression of F-actin protein indicates a regular distribution of cells, following the treatment with SH, which leads to a well-organized structural continuity of the epithelial barrier. These data indicate that SH has a beneficial impact on skin barrier, improving filaggrin expression, which is essential for the correct formation and function of the skin.

### Metalloproteinase expression

Skin exposure to ultraviolet radiations, induces impairments in the extracellular matrix (ECM) causing skin wrinkling, sagging, and laxity, the major characteristics of skin aging. Matrix metalloproteinases (MMPs) are the principal responsible for degrading the ECM components such as collagen, elastin, fibronectin, and proteoglycans [33]. A large amount of studies underlines that skin exposure to UV irradiation induces upregulation of different MMPs, impacting skin appearance and health [34–37].

**Figure 10:**
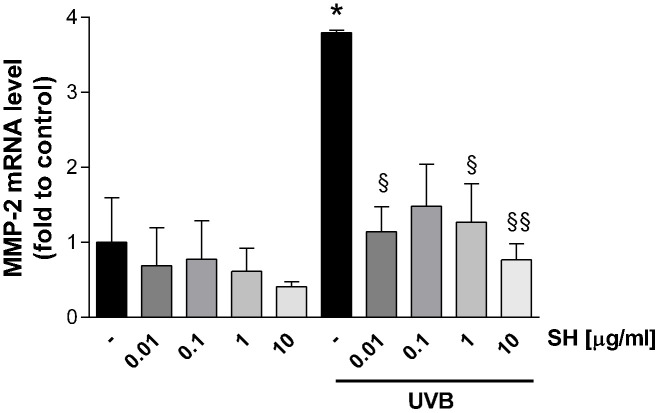
**Effect of *Salvia haenkei* extract 0.01-0.1-1-10 μg/ml on MMP-2 mRNA levels in HaCaT cells exposed and not exposed to UVB radiation (30 KJ/m^2^).** Results are normalized to GAPDH, expressed as ratio of treatment *vs* control not exposed to UVB, and are the mean ± SEM of 3 independent experiments. *p < 0.05, treatment *vs* control-UVB; § *p* < 0.05, §§ *p* < 0.01, treatment *vs* control+UVB.

Results in Figure 10 show that UVB radiation increases the mRNA expression of the gelatinase MMP-2 which degrades basement membrane collagens and denatured structural collagens [38]. Treatment with SH is able to significantly reduce MMP-2 mRNA levels (SH 0.01-0.1-10 μg/ml) indicating activity of SH extract in the maintenance of the structural integrity of the skin, damaged by photo-stress.

## DISCUSSION

Skin aging is the cumulative effect of intrinsic and extrinsic factors which lead to a decline in skin, inducing thinning, stiffing, and loss of flexibility. Skin is the first barrier between the organism and environment, so the maintenance of its functional and anatomical integrity is essential. Besides natural aging processes and aging-related conditions such as diabetes, menopause, etc., extrinsic factors can concur in affecting skin structure; in particular photodamage, pollution or lifestyle factors (smoking, diet, emotional stress, etc.) may hamper skin structure and functions [39, 40]. Keratinocytes are the major constituents of the epidermis, providing the cellular basis for the outermost barrier; in this work, HaCaT human keratinocyte cell line has been exposed to stress factors, such as free radicals and ultraviolet B radiation, in order to mimic the natural skin-aging, thus altering the barrier function. *Salvia haenkei* has been recently identified as a potential anti-senescence agent suggesting its possible application in skin aging and skin aging-related disorders; therefore, the effect of an extract of this plant was deeply investigated on parameters related to skin ageing, maintenance of skin integrity and physiological conditions. The production of reactive oxygen species or free radicals through normal endogenous metabolic processes or UVR and pollution is thought to contribute to skin aging processes. Skin is vulnerable to a variety of redox reactions, and the redox balance is essential in the maintenance of skin homeostasis. Low ROS concentrations exert physiological activities, but increased levels of these species are involved in the activation of pathways resulting in increased degradation of collagen, accumulation of elastin, activation of matrix-degrading metalloproteases, etc. [41]. Moreover, ROS play a significant role in pathological processes including aging, inflammation, injuries, and pathogenesis of several skin diseases such as psoriasis and vitiligo [42]. Interestingly, *Salvia haenkei* extract has been shown to reduce ROS levels in basal conditions, in oxidative stress condition, and in stress conditions induced by UVB irradiation, indicating a potential protective effect against ROS mediated alterations [22].

In support of the previously observed antiaging effect of *Salvia haenkei*, in this work, we evidenced the ability of SH extract to reduce senescence in keratinocytes stimulated by UVB radiation. Results show that SH is able to reduce the gene expression of p21 and p27 senescence markers, which is stimulated by UVB radiation. Interestingly, SH extract shows promising activity in reducing the inflammatory response fostered by photodamage in cells, by lowering interleukins transcription. Furthermore, we have intriguingly demonstrated that *Salvia haenkei* extract induced Sirtuin1 protein expression not only after stress but also under basal conditions, showing promising activity in slowing down aging processes. Sirtuin family, and in particular SIRT1, has been shown to play a role in aging-photoaging, especially through the inhibition of MMPs and subsequent collagen degradation; multiple studies underlined its protective role in UVB-mediated photoaging. Natural compounds like juglone (5-hydroxy-1,4-napthalenedione) have been shown to restore SIRT1 to normal levels after UVB treatment, suggesting that SIRT1 might play a role in preventing UVB-induced carcinogenesis [29]. Our results indicate as well, a possible involvement of sirtuins in the repairing process after UVB-stress and their role in the maintenance of epidermis homeostasis.

Common inflammatory skin disorders, such as atopic dermatitis and psoriasis, as well as aging processes, exhibit decreased barrier function, and the response of epidermal cells to barrier disruption may aggravate, maintain, or even initiate such conditions. The results of this work revealed a strong ability of SH to restore the skin barrier integrity by modulation of several proteins involved in these processes. In particular, it was observed a stimulation of occludin and filaggrin transcription indicating a reinforcement action on adhesions between cells. Inherited and acquired filaggrin deficiency affects the epidermal barrier, altering the organization of cytoskeleton keratin filaments and the structure of the cornified envelope of the skin [43]. Furthermore, defects in FLG expression induce a decrease in the number of keratohyalin granules, a marked fall in natural moisturizing factor (NMF) concentration, and alkalinization of the skin pH [44]. Moreover, alterations in claudins and occludin proteins, which belong to the Tight junctions (TJs) proteins, are well described concerning aging processes. TJs are responsible for sealing the intercellular space between epithelial cells, to form a functional barrier, and are involved in cell proliferation and differentiation [45]. *Salvia haenkei* extract has been shown to improve filaggrin expression and to preserve the occludin localization on cell membranes, impaired by UVB radiations. An additional activity of SH extract in the maintenance of healthy skin has been evidenced by its ability to reduce matrix metalloproteinases 2 (MMP-2) transcription, stimulated by UVB radiation. The upregulation of MMPs facilitates aging and skin pathologies, by degrading collagen and elastin causing impairments in the extracellular matrix (ECM) [33, 38], thus a reduction of these proteinases is worthful in the context of skin aging.

The positive effects of SH treatment observed in this study over the most common skin molecular parameters were confirmed also by a recent clinical trial, executed in a cohort of fifty Caucasian women aged between 45 and 60 [46]. *Salvia haenkei* has been administered in a cream formulation and several parameters, such as skin redness and elasticity, wrinkle depth and antioxidant potential, were evaluated by both clinical and instrumental evaluations as well as self-assessment by subjects. Results of this study reveal SH efficacy in ameliorating the skin decline when applied daily in a context of moderate skin ageing. In fact, after 84 days of application, the women’s skin resulted to be more elastic and presented a lower number of red areas and shallower wrinkles. Moreover, the study demonstrates a progressive increase in skin antioxidant capability following daily application of the cream [46].

As a protective shelter for the body from the external environment, the skin is constantly exposed to potential injury, and thus wound healing is an essential process for the survival of all organisms. Considering the proven ability of SH in maintaining healthy and unblemished skin, we assumed that this extract could help to also enhance skin regeneration being useful in the repair of skin lesions. Thus, SH activity on keratinocytes migration, being a key step of restoring the skin barrier after injuries, was finally considered. SH extract treatment revealed an interesting activity being able to increase HaCaT speed of migration, demonstrating a strong effect on the re-establishment of epithelial injury after lesions. As migration is a critical element in the wound healing process, our data, also considering the non-hyperproliferative activity of the extract, indicate a potential effect of SH extract in faster counteracting the effect of an injury in the skin barrier, reducing the risk of infections.

Our data points out *Salvia haenkei* extract as a useful compound for either aiding barrier re-establishment or dampening the epidermal stress response. Concomitantly with the recent trial in humans, this work furnishes further insights into the mechanisms involved in the positive effect of the extract in skin aging.

This work, in support to the previous works [18, 46], identifies in a more peculiar way molecular mechanism linked to the efficacy of *Salvia haenkei* in modulating skin parameters, underlining the relevance of *Salvia haenkei* as an appealing agent useful for anti-ageing and ageing-related skin disorders.

## MATERIALS AND METHODS

### Cell culture and UVB irradiation

HaCaT cells (human spontaneously immortalized keratinocytes) were cultured in high glucose Dulbecco's modified Eagle's medium (DMEM) supplemented with 10% fetal bovine serum Life-Technologies, Waltham, MA, USA), 2 mM glutamine, 100 U/ml penicillin and 100 μg/ml streptomycin (Lonza, Basilea, Switzerland). Cells were maintained under a humidified atmosphere of 5% CO_2_ in air and incubated at 37° C.

When requested by the experimental protocol, cells were exposed to 30 KJ/m^2^ UVB radiation, supplied by a Philips Medical Narrowband UVB PL-S lamp. To prevent light absorption by tissue-culture medium, this was removed prior to irradiation, and replaced with DMEM FBS and red phenol free, following the irradiation protocol [47]. After UVB irradiation, cells were fed with fresh growth medium or used for experiment following the protocol.

### Cell viability - Trypan blue exclusion assay

35 x10^3^ cells were plated on 12 well plates and, following overnight incubation, were exposed to different concentrations of *Salvia haenkei* hydroethanolic extract according to experimental protocols. The extract has been kindly provided by IBSA Farmaceutici Italia (Lodi, Italy) and presents a content of the marker rosmarinic acid of 0.6% w/w by HPLC. After treatment, cells were washed, detached with 0.25% trypsin-0.2% EDTA and suspended in trypan blue (Sigma-Aldrich, St Louis, MO, USA) at 1:1 ratio in medium solution. Cells were counted using a chamber Burker hemocytometer.

### ROS fluorescence assay

ROS were quantified using 2′,7′-dichlorofluorescin-diacetate (H_2_-DCF-DA, Sigma-Aldrich, St Louis, MO, USA), as previously described [48]. Briefly, cells (5x10^3^) were seeded into 96-well plates, allowed to adhere overnight and then exposed to SH extract for 3 or 24 hours. N-acetylcysteine 5mM (Sigma-Aldrich, St Louis, MO, USA) was used as positive control.

For the oxidative stress condition, the ROS level was measured after addition of 50 μM H_2_-DCF-DA in absence or presence of H_2_O_2_ [48]. DCF fluorescence intensity was measured at excitation 485 nm—emission 535 nm, using a Multilabel Plate Reader VICTOR X3 (PerkinElmer, Waltham, MA, USA).

For the evaluation of ROS levels in UVB stress conditions, the protocol was slightly modified, by incubating cells in SH treatments - 50 μM H_2_-DCF-DA solution in PBS. Following 25 minutes of incubation at 37° C, the plate was exposed to UVB radiations and the DCF fluorescence intensity was immediately measured at excitation 485 nm—emission 535 nm, using a Multilabel Plate Reader VICTOR X3 (PerkinElmer, Waltham, MA, USA).

Fold increase in ROS production was calculated using the equation: $\left(F_{treatment}-F_{blank}|F_{control}-F_{blank}\right)$, where F is the fluorescence reading.

### RNA expression/quantitative real-time PCR

10x10^4^cells were seeded into 6-well plates, and, following overnight incubation, were exposed to SH extract for 24 hours. UVB stimulation was performed as already described, and SH treatment in complete medium was applied for another 16 hours.

RNA was isolated with the TRIzol method (Invitrogen, Thermo Fisher Scientific, Waltham, MA, USA) and retro-transcription was performed with High-Capacity cDNA Reverse Transcription Kit (Applied Biosystems, Thermo Fisher Scientific, Waltham, MA USA) according to the manufacturer’s instructions. Quantitative PCR (qPCR) reactions were performed with a QuantStudio™ 5 (Invitrogen) using Power SYBR™ Green PCR Master Mix (Applied Biosystems, Thermo Fisher Scientific, Waltham, MA USA) and the specific primers reported below.

Primer sequences were obtained from PrimerBank (http://pga.mgh.harvard.edu/primerbank/index.html).

GAPDH expression level was used as a reference for the normalization of each value level. The primer sequences used were as follows: p21 forward, 5’-TGTCCGTCAGAACCCATGC-3’; reverse, 5’-AAAGTCGAAGTTCCATCGCTC-3’, p27 forward, 5’-TAATTGGGGCTCCGGCTAACT-3’; reverse, 5’-TGCAGGTCGCTTCCTTATTCC-3’; IL-1α forward, 5’-GACGGTTGAGTTTAAGCCAATCC-3’; reverse 5’-CAGGAAGCTAAAAGGTGCTGAC-3’; IL-6 forward, 5’-TACATCCTCGACGGCATCTC-3’; reverse, 5’-TGCCTCTTTGCTGCTTTCAC-3’. IL-18 forward, 5’-CCAAGGAAATCGGCCTCTATTTG-3’; reverse, 5’-ATATGGTCCGGGGTGCATTATC-3’; MMP2 forward, 5’-TACAGGATCATTGGCTACACACC-3’; reverse, 5’-GGTCACATCGCTCCAGACT-3’; GAPDH forward 5’-AATCCCATCACCATCTTCCA-3’; reverse, 5’- TGGACTCCACGACGTACTCA-3’.

### Immunoblot assay

6-multiwell plates were seeded with 75x10^3^ cells and incubated overnight. Cells were then treated according to the protocol and then lysed with ice-cold lysis buffer, supplemented with the protease inhibitor cocktail (Roche Molecular Biochemicals, Mannheim, Germany). Cell lysates were then centrifuged at 14000 rpm for 15 minutes at 4° C and the supernatant protein content was determined by the Lowry procedure (Bio-Rad DC Protein Assay, MA, USA).

Equal amount of protein was loaded on a polyacrylamide gel and electrophoretically separated in running buffer. After electrophoresis, the proteins were blotted onto a Hybond-P PVDF membrane (Amersham Biosciences, Buckinghamshire, UK). After blocking with a 10% skim milk solution, the membrane was exposed to the primary antibody anti-SIRT1 (1:250; Sigma-Aldrich, St Louis, MO, USA), and, following overnight incubation, was washed and exposed to HRP-conjugated anti-rabbit secondary antibody (1:3500; PerkinElmer, MA, USA). The signal was visualized with an enhanced chemoluminescent kit (Amersham Biosciences, Buckinghamshire, UK) according to the manufacturer's instructions and analyzed by Molecular Imager VersaDoc MP 4000 (Bio-Rad, Hercules, CA, USA). All the proteins were normalized to calnexin (1:1000; Santa Cruz Biotechnology INC, Santa Cruz, CA, USA).

### Wound healing assay

10x10^4^ cells were seeded in 4-well culture chambers with glass bottom (Sarstedt AG & Co, Nümbrecht, Germany) pre-coated with collagen. A two-wells silicone insert with a defined cell-free gap was used to mechanically form a cell-free area. Once reached the surface confluence, the silicon insert was removed, and cells were treated with SH extract 0.01 and 0.1 μg/ml. The HaCaT migration in the cell free gap was recorded for 24 hours using an in live cells time lapse confocal microscope LSM 800 software ZEN 2.1 blue edition (Carl Zeiss, Jenza, Germany), magnification 20X. Data were analyzed with the dedicated software.

### Immunofluorescence microscopy

10x10^4^ cells were seeded on glass coverslips precoated with collagen in 24-well plates, allowed to attach overnight, and treated with SH extract according to protocol. For detection of β-catenin and occludin proteins, cells were exposed to UVB radiation, as described in the protocol above. After irradiation, cells were fed with fresh growth medium or used for experiment following the protocol. For immunofluorescence assay, 4 hours after the UVB irradiation, cells were washed, fixed with 4% formaldehyde, permeabilized with 0.1% Triton X-100 in PBS and stained for 1 hour at 37° C with different protein-specific antibody: mouse monoclonal anti-occludin Invitrogen Life Technologies, Waltham, MA, USA mouse anti-filaggrin (Sigma-Aldrich, St Louis, MO, USA). After PBS wash, cells were incubated with secondary antibodies/fluorescein isothiocyanate (Alexa Fluor 488 anti-mouse or Alexa Fluor 536 anti-rabbit immunoglobulin G, Molecular Probes, Invitrogen Life Technologies, Waltham, MA, USA) and with phalloidin for 1 hour at room temperature. Following 10 minutes of treatment with RNase, the coverslips were mounted on glass slides by using Mowiol 40–88 (Sigma-Aldrich, St Louis, MO, USA) added of propidium iodide. Images were acquired through confocal microscope LSM 800, magnification 60X, software ZN 2.1 blue Edition (Carl Zeiss, Jenza, Germany) and quantified using ImageJ software.

### Statistical analysis

The statistical analysis was performed using GraphPad Prism version 7.02 for Windows (GraphPad Software, San Diego, CA, USA). Unless stated otherwise, results are presented as mean ± SEM. The Student’s *t*-test was used and *p* values < *0.05* were considered as statistically significant.
